# The Succession of the Gut Microbiota in Insects: A Dynamic Alteration of the Gut Microbiota During the Whole Life Cycle of Honey Bees (*Apis cerana*)

**DOI:** 10.3389/fmicb.2021.513962

**Published:** 2021-04-14

**Authors:** Zhi-Xiang Dong, Yi-Fei Chen, Huan-Yuan Li, Qi-He Tang, Jun Guo

**Affiliations:** Faculty of Life Science and Technology, Kunming University of Science and Technology, Kunming, China

**Keywords:** *Apis cerana*, core microbiota, colonization, relative abundance, environmental exposure

## Abstract

The Asian honey bee *Apis cerana* is a valuable biological resource insect that plays an important role in the ecological environment and agricultural economy. The composition of the gut microbiota has a great influence on the health and development of the host. However, studies on the insect gut microbiota are rarely reported, especially studies on the dynamic succession of the insect gut microbiota. Therefore, this study used high-throughput sequencing technology to sequence the gut microbiota of *A. cerana* at different developmental stages (0 days post emergence (0 dpe), 1 dpe, 3 dpe, 7 dpe, 12 dpe, 19 dpe, 25 dpe, 30 dpe, and 35 dpe). The results of this study indicated that the diversity of the gut microbiota varied significantly at different developmental stages (ACE, *P* = 0.045; Chao1, *P* = 0.031; Shannon, *P* = 0.0019; Simpson, *P* = 0.041). In addition, at the phylum and genus taxonomic levels, the dominant constituents in the gut microbiota changed significantly at different developmental stages. Our results also suggest that environmental exposure in the early stages of development has the greatest impact on the gut microbiota. The results of this study reveal the general rule of gut microbiota succession in the *A. cerana* life cycle. This study not only deepens our understanding of the colonization pattern of the gut microbiota in workers but also provides more comprehensive information for exploring the colonization of the gut microbiota in insects and other animals.

## Introduction

The gut microbiota has attracted extensive attention due to its close relationship with the host. The gut microbiota not only promotes digestion and absorption of food by the host (Cummings, 1984) but also plays an important role in host development (Sommer and Bäckhed, 2013; Lin et al., 2019), immunity (Pickard et al., 2017), aging (O’Toole and Jeffery, 2015), and resistance to pathogen invasion (Pamer, 2016). The realization of gut microbiota function depends on the composition and structure of the gut microbiota. The composition and structure of the gut microbiota change dynamically during the whole life cycle of the host. Interestingly, previous studies have shown that improving the structure of the host gut microbiota can increase the lifespan of the host (Smith et al., 2017; Han et al., 2018). For example, by transferring young African turquoise killifish (*Nothobranchius furzeri*) gut microbes into older hosts, the lifespan of the hosts was increased, and the rate of decline in exercise ability was delayed (Smith et al., 2017). In addition, the rapid change in the abundance of subdominant bacteria in the gut is a hallmark of human aging. Interestingly, health-related bacteria were enriched in a long-lived population (Biagi et al., 2016).

There are few reports on the colonization and succession of the host gut microbiota in the natural state, and most research has focused on vertebrates, such as foals (Costa et al., 2016), goats (Lei et al., 2018), chickens (Xi et al., 2019), and southern catfish (Zhang et al., 2018). These results indicate that the structure of the gut microbiota is constantly changing with the development of the host, and the structure of the gut microbiota is significantly correlated with host age. These studies not only revealed the succession of a series of gut microbiota species but also provided an important reference value for studying the colonization of other gut microbiota species.

Insects (Arthropoda: Insecta) are the most ubiquitous and diverse animals on the planet. The relationship between the composition of insect gut microbes and the host has gradually been revealed, including gut microbes with effects on host immunity (Wei et al., 2017), metabolism (Zheng et al., 2017), environmental exposure (Wintermantel et al., 2018), and pest control (Xie et al., 2019). In addition, the succession of insect gut microbes has also been studied. For example, Duguma et al. (2015) found that the *Culex* mosquito gut microbiota had different structures in different stages of development. Similar studies have been conducted in other insects, such as the burying beetle (*Nicrophorus vespilloides*) (Wang and Rozen, 2017), the cockroach (*Blattella germanica*) (Purificación et al., 2014), the queen bee (*Apis mellifera*) (Tarpy et al., 2015) and *Drosophila melanogaster* (Han et al., 2017). The results of these studies all revealed that the structure of the gut microbiota showed a significant correlation at different stages of development in different insects. However, little is known about the natural succession of the insect gut microbiota in the natural state, such as that in honey bees.

Honey bees are important pollinators and convey great economic benefits to crop pollination worldwide (Southwick and Southwick, 1992; Kevan, 1999; Klein et al., 2007; Kleijn et al., 2015). In addition, the relationship between the bee gut microbiota and health has received considerable attention. When honey bees are exposed to pesticides, their gut microbiota is disturbed, and their mortality increases (Motta et al., 2018). Moreover, the overuse of antibiotics also leads to structural changes in the gut microbiota of honey bees, making them more susceptible to infection by pathogens and leading to greater challenges to bee survival (Raymann and Moran, 2017). In addition, when *A. cerana* were infected with *Nosema ceranae*, the steady state of the gut microbiota was disturbed, leading to an increase in the mortality rate of the bees (Huang et al., 2018). These studies all suggest that the gut microbiota plays an important role in bee health and disease. However, the gut microbes of *A. cerana* are poorly studied, and little is known about the succession rules of the gut microbiota of honey bees. This lack of knowledge limits our understanding of the gut microbiota throughout the life cycle of *A. cerana* and hinders our ability to protect resource insects.

The goal of this study was to elucidate the dynamic changes in the gut microbiota throughout the life cycle of *A. cerana* from the perspective of the composition and structure of the gut microbiota. In this study, high-throughput sequencing technology was used to sequence the gut microbiota of workers 0 days post emergence (dpe), 1 dpe, 3 dpe, 7 dpe, 12 dpe, 19 dpe, 25 dpe, 30 dpe, and 35 dpe. The purpose of this study was to elucidate the colonization rules of gut microbes in *A. cerana*, which inform important theories for improving host health through gut microbes. In addition, this study provides reference information for the study of gut microbe colonization in other animals.

## Materials and Methods

### Worker Sampling

Worker samples were collected in Kunming, Yunnan Province in July 2018. To obtain bees of different ages, we conducted sample collection in accordance with the method described in previous publications (Powell et al., 2014; Guo et al., 2015). First, we identified a healthy hive, determined the age of the workers and selected a frame in which new workers were appearing. A frame of late-stage pupae (eyes were pigmented, but pupae lacked movement) was selected from the hive and moved to a sterile incubator (34°C and 90% relative humidity, mimicking hive conditions), and the pupae were allowed to eclose naturally. We first collected worker samples 0 dpe (0 days post emergence) and placed them in 1.5 mL centrifuge tubes (0 dpe individuals had no contact with the environment). Next, 300 newly emerged worker individuals from the incubator were marked with red Testors enamel paint and then returned to the original hive to allow for natural growth. Finally, according to the life cycle of the bees, samples were randomly collected 1 dpe, 3 dpe, 7 dpe, 12 dpe, 19 dpe, 25 dpe, 30 dpe and 35 dpe. Three worker samples were collected 0 dpe, 1 dpe, 3 dpe and 7 dpe, and six worker samples were collected 12 dpe, 19 dpe, 25 dpe, 30 dpe and 35 dpe. All worker samples were immediately placed in an ultralow temperature freezer (EU1DW/BD-55W321EU1, China) after collection.

In the process of sample collection, we to reduce the contamination of the samples, including the use of sterile centrifuge tubes. All experiments were conducted on a sterile ultraclean platform, and all the equipment was treated with high-temperature sterilization.

### DNA Extraction and PCR Amplification

First, workers were removed from the ultralow temperature freezer and placed onto an ultraclean working table. Then, sterile tweezers were used to remove the entire gut of the workers, and the gut was then placed into a 1.5 mL sterile centrifuge tube. Then, 60 μL of Krebs Ringer buffer was added to the centrifuge tube, and the sample was ground. A soil DNA kit (Omega Biotek, Norcross, GA, United States) was used to extract the gut bacterial DNA of the workers according to the manufacturer’s protocol. Finally, 60 μL of elution buffer was added to obtain the DNA sample, and the resulting DNA sample was stored in a freezer at −20°C. The final DNA concentration and purity were determined with a NanoDrop 2000 UV-vis spectrophotometer (Thermo Scientific, Wilmington, United States), and the DNA quality was determined with 1% agarose gel electrophoresis. The V3-V4 hypervariable regions of the bacterial 16S rRNA gene were amplified with the primers 338F (5′-ACTCCTACGGGAGGCAGCAG-3′) and 806R (5′-GGACTACHVGGGTWTCTAAT-3′) by a thermocycler PCR system (GeneAmp 9700, ABI, United States). PCR was conducted using the following program: 3 min of denaturation at 95°C; 27 cycles of 30 s at 95°C, 30 s annealing at 55°C, and 45 s elongation at 72°C; and a final extension at 72°C for 10 min. Each PCR was performed in triplicate in 20 μL of reaction mixtures containing 4 μL of 5 × FastPfu buffer, 2 μL of 2.5 mM dNTPs, 0.8 μL of each primer (5 μM), 0.4 μL of FastPfu Polymerase and 10 ng of template DNA. The resulting PCR products were extracted from a 2% agarose gel, further purified using an AxyPrep DNA gel extraction kit (Axygen Biosciences, Union City, CA, United States) and quantified using QuantiFluor^TM^-ST (Promega, United States) according to the manufacturer’s protocol.

### Illumina MiSeq Sequencing and Processing of Sequencing Data

Purified amplicons were pooled in equimolar amounts and paired-end sequenced (2 × 300) on an Illumina MiSeq platform (Illumina, San Diego, United States) according to the standard protocols by Majorbio Bio-Pharm Technology Co., Ltd. (Shanghai, China).

MiSeq sequencing results were reported as paired-end sequence data. First, according to the overlap relation between PE reads, pairs of reads were merged into a single sequence. At the same time, the quality of the reads and the effect of merging were used as filters. Barcode and primer sequences at both ends of the sequence were used to distinguish the samples and obtain the effective sequence. In addition, the sequence direction was corrected to optimize the data.

Raw fastq files were quality filtered by Trimmomatic and merged by FLASH with the following criteria: (i) The reads were truncated at any site with an average quality score <20 over a 50 bp sliding window; (ii) sequences with overlaps longer than 10 bp were merged according to their overlap, with no more than 2 bp mismatched; and (iii) the sequences of each sample were separated according to barcodes (exactly matching) and primers (allowing 2 nucleotide mismatches), and reads containing ambiguous bases were removed. Operational taxonomic units (OTUs) were clustered with a 97% similarity cutoff using UPARSE (version 7.1)^1^ with a novel “greedy” algorithm that performs chimera filtering and OTU clustering simultaneously. The taxonomy of each 16S rRNA gene sequence was analyzed by the RDP Classifier algorithm^2^ against the Silva (SSU123) 16S rRNA database at a confidence threshold of 70%.

### Statistical Analyses and Comparison of Microbial Communities

One-way analysis of variance (ANOVA) was used to analyze the diversity parameters (ACE, Chao1, Shannon and Simpson) of the gut microbiota of workers at different dpe (the time points after the emergence of workers, 0 dpe, 1 dpe, 3 dpe, 7 dpe, 12 dpe, 19 dpe, 25 dpe, 30 dpe and 35 dpe). In addition, Kruskal-Wallis H test was performed to analyze the bacteria with the highest abundance (phylum and genus taxa) in the gut of workers at different dpe. According to the gut microbiota abundance of workers of different ages, Kruskal-Wallis H test was performed to conduct hypothesis tests for different groups. In addition, the significance level of differences in species abundance was evaluated, and the species with significant differences at different ages were obtained (from analysis using the stats package in R and the SciPy package in Python). The non-metric multidimensional scaling (NMDS) distance algorithm based on Bray-Curtis distance was used to calculate the differences between the assessment of microbial communities (QIIME was used to calculate the beta diversity distance matrix, and the R language vegan software package was used for NMDS analysis and mapping).

## Results

### Summary of the Sequencing Data

Deep sequencing of 42 gut samples from workers yielded 1,988,217 sequences with a total length of 886,569,845 bp and an average length of 445.91 bp. A total of 1670 OTUs were obtained at a 97% similarity level. Cluster analysis was conducted at the phylum and genus levels, and 30 phyla and 512 genera were obtained. Good’s coverage index showed that the estimated values of all samples were over 99%, indicating that all samples reached an appropriate sequencing depth (Table 1). In addition, the raw reads were deposited into the NCBI Sequence Read Archive (SRA) database (accession numbers SRR9715685-SRR9715699, SRR9715700-SRR9715726).

**TABLE 1:** Richness and diversity indices relative to each gut sample.

**Sample name**	**Coverage**	**Alpha diversity**			
		**Ace^ab,bc^**	**Chao^ab,bc,^**	**Shannon^ab,bc^**	**Simpson^ab^**
0 dpe1^a^	0.9997	211.70	208.06	2.25	0.31
0 dpe2	0.9996	228.82	243.00	2.71	0.20
0 dpe3	0.9997	277.04	277.58	2.72	0.23
1 dpe1^b^	0.9987	951.04	959.05	4.85	0.04
1 dpe2	0.9993	743.14	755.33	4.87	0.04
1 dpe3	0.9996	605.84	613.33	3.99	0.13
3 dpe1	0.9984	487.49	484.50	1.87	0.36
3 dpe2	0.9981	841.55	847.51	3.47	0.18
3 dpe3	0.9996	419.22	420.71	2.16	0.46
7 dpe1^c^	0.9983	390.13	386.23	2.08	0.19
7 dpe2	0.9985	485.01	484.51	2.32	0.17
7 dpe3	0.9990	465.36	478.69	3.10	0.11
12 dpe1	0.9975	427.56	407.17	1.98	0.23
12 dpe2	0.9989	224.41	196.08	1.93	0.18
12 dpe3	0.9977	434.01	405.45	2.00	0.21
12 dpe4	0.9971	524.17	525.26	1.82	0.26
12 dpe5	0.9973	599.82	496.44	2.13	0.20
12 dpe6	0.9981	408.75	398.33	2.36	0.17
19 dpe1^d^	0.9966	533.32	534.85	2.00	0.24
19 dpe2	0.9985	360.22	363.63	2.13	0.25
19 dpe3	0.9977	617.02	611.49	2.99	0.10
19 dpe4	0.9983	369.31	365.61	2.01	0.26
19 dpe5	0.9979	401.82	311.86	1.72	0.23
19 dpe6	0.9983	338.69	337.50	1.48	0.37
25 dpe1	0.9989	189.24	181.04	1.26	0.42
25 dpe2	0.9989	233.72	177.45	1.77	0.25
25 dpe3	0.9983	259.59	255.16	2.17	0.20
25 dpe4	0.9976	494.64	358.35	1.77	0.27
25 dpe5	0.9959	607.74	618.13	2.51	0.15
25 dpe6	0.9975	443.40	402.00	2.03	0.20
30 dpe1^e^	0.9969	914.48	922.20	3.79	0.07
30 dpe2	0.9997	572.88	579.14	5.26	0.02
30 dpe3	0.9984	240.18	202.67	1.58	0.30
30 dpe4	0.9970	523.16	497.87	1.94	0.26
30 dpe5	0.9969	492.45	502.20	2.09	0.20
30 dpe6	0.9987	388.42	382.16	1.98	0.22
35 dpe1	0.9984	304.89	307.56	1.69	0.30
35 dpe2	0.9990	169.79	159.04	1.76	0.24
35 dpe3	0.9987	199.77	175.03	1.87	0.19
35 dpe4	0.9975	484.45	389.12	1.79	0.23
35 dpe5	0.9962	855.79	878.00	3.76	0.06
35 dpe6	0.9977	387.62	354.16	2.03	0.24
*P* value		0.045	0.031	0.0019	0.041

*^*ab*^Shows that the diversity index was significantly different between 0 dpe and 1 dpe.*

*^*bc*^Shows that the diversity index was significantly different between 1 dpe and 7 dpe.*

### Changes in Gut Microbiota Diversity in Workers of Different Ages

The diversity of bacterial communities is reflected through the Shannon and Simpson indices, and the richness of the community is reflected by the Chao1 and ACE indices. The results showed that the diversity of the gut microbiota was influenced by the age of the host; moreover, the composition and structure of the gut microbiota of workers of different ages were significantly different (Table 1). Namely, the *P*-values of the bacterial diversity index differences were as follows: ACE, *P* = 0.045; Chao1, *P* = 0.031; Shannon, *P* = 0.0019; and Simpson, *P* = 0.041.

### Composition of the Gut Microbiota in Workers of Different Ages

At the phylum level of classification, the gut microbiota of workers consisted mainly of the following bacteria: Proteobacteria, Cyanobacteria, Planctomycetes, Spirochaetae, Bacteroidetes, Actinobacteria, Verrucomicrobia, Acidobacteria, Firmicutes, and Fibrobacteres. The results of this study also showed that there were significant differences in the relative abundance of some of the top 10 phyla in the gut of workers of different ages (Table 2). Proteobacteria had the highest relative abundance 0 dpe (71.97%) and the lowest abundance 1 dpe (15.50%) (*P* = 0.013). Bacteroidetes are an important and consistent components of the worker gut, and their relative abundance was lowest 0 dpe (2.45%) and highest 30 dpe (28.07%) (*P* = 0.021). Actinobacteria had the highest relative abundance 0 dpe (11.06%) and the lowest 1 dpe (0.72%). In addition, the relative abundance of Firmicutes was lowest 0 dpe and highest 3 dpe (54.94%) (*P* = 0.008). Moreover, the following bacteria were significantly different in the gut of workers of different ages: the relative abundance of cyanobacteria was highest at 1 dpe and lowest at 25 dpe (*P* = 0.015), the relative abundance of Spirochaetae was highest 1 dpe and lowest at 25 dpe (*P* = 0.0095), the relative abundance of Fusobacteria was lowest 0 dpe and highest 1 dpe (*P* = 0.011), the relative abundance of Verrucomicrobia was lowest 0 dpe and highest 1 dpe (*P* = 0.032), the relative abundance of Acidobacteria was highest 0 dpe and lowest at 25 dpe (*P* = 0.00061), and the relative abundance of Chloroflexi was highest 0 dpe and lowest 19 dpe (*P* = 0.0092) (Supplementary Figure 1).

**TABLE 2:** Top 10 abundant phyla.

**Phylum**	**Different post time (%)**									***P* value**
	**0 dpe^a^**	**1 dpe^b^**	**3 dpe**	**7 dpe^c^**	**12 dpe**	**19 dpe^d^**	**25 dpe**	**30 dpe^e^**	**35 dpe**	
Proteobacteria^ab^	71.97	15.50	26.73	22.21	29.58	37.75	48.01	40.61	46.77	0.013
Cyanobacteria	1.44	2.33	2.13	0.91	0.85	0.66	0.11	1.42	0.56	0.015
Planctomycetes	0.03	0.01	0.01	0.00	0.00	0.00	0.00	0.00	0.00	0.172
Spirochaetae^bc^	0.30	4.62	1.48	0.60	0.15	0.27	0.14	1.59	0.50	0.010
Bacteroidetes^ab,bc^	2.45	23.58	8.91	12.02	18.51	17.75	24.43	28.07	23.09	0.021
Actinobacteria^ab^	11.06	0.72	4.11	12.07	1.68	3.66	0.77	1.99	1.83	0.067
Verrucomicrobia^bc^	0.03	0.94	0.32	0.06	0.04	0.03	0.03	0.29	0.08	0.032
Acidobacteria	0.31	0.04	0.02	0.00	0.00	0.00	0.00	0.00	0.00	0.001
Firmicutes^ab^	5.26	47.40	54.94	51.34	48.98	39.59	26.30	23.73	26.35	0.008
Fibrobacteres	0.00	0.10	0.05	0.01	0.00	0.01	0.01	0.06	0.01	0.013
Others	7.10	4.66	1.27	0.75	0.20	0.28	0.20	2.18	0.81	

*^*ab*^Indicates that the relative abundance 0 dpe and 1 dpe at two developmental stages is significantly different (Student’s *t* test).*

*^*bc*^Indicates that the relative abundance 1 dpe and 7 dpe at two developmental stages is significantly different (Student’s *t* test).*

**FIGURE 1:**
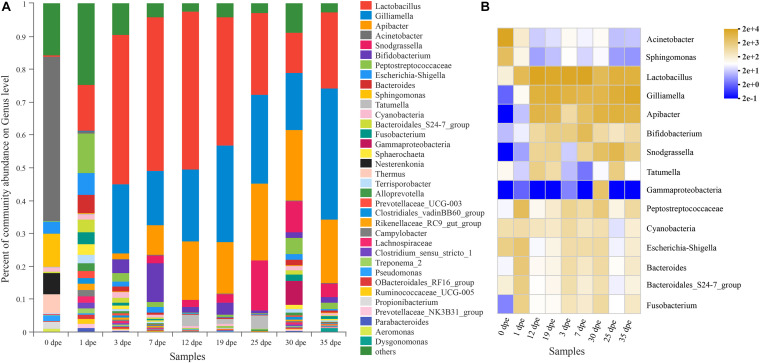
**(A)** The abscissa is the sample name, and the ordinate is the proportion of species in the sample. The column of different colors represents different species, and the length of the column represents the proportion of the species. **(B)** The abscissa is the name of the sample, and the ordinate is the name of the species. The abundance of different species in the sample is shown by the color gradient of the color block. The right side of the figure is the value represented by the color gradient.

The results of this study indicated that there were 35 genera with a relative abundance higher than 1% in the gut of *A. cerana* workers (Figure 1A). Among them, the genera with the highest relative abundances were *Lactobacillus*, *Gilliamella*, *Apibacter*, *Acinetobacter*, *Snodgrassella*, *Bifidobacterium*, *Peptostreptococcaceae*, *Escherichia-Shigella*, *Bacteroides*, and *Sphingomonas*. In addition, the gut microbiota 0 dpe and 1 dpe had similarities in structure, and the genera with the highest relative abundances were *Acinetobacter*, *Sphingomonas* and *Lactobacillus* (Figure 1B). After 3 dpe, the structure of the gut microbiota was more uniform (Figure 1B).

**FIGURE 2:**
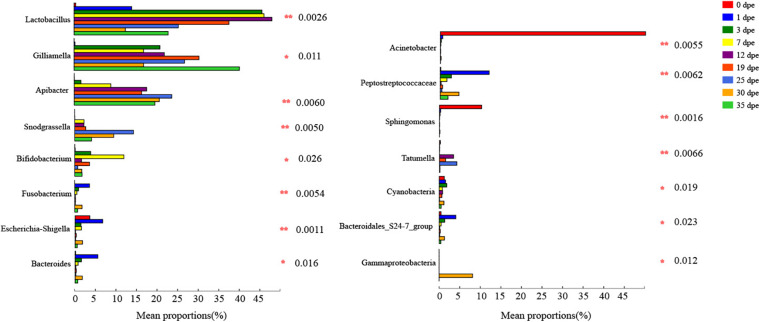
The vertical axis represents the species names at genus classification level, the corresponding column length represents the average relative abundance of the species in various groups, and different colors represent different groups. On the far right is the value of *P*, *0.01 < *P* ≤ 0.05, **0.001 < *P* ≤ 0.01, ***P ≤ 0.001.

An interesting result of this study was that there were significant differences in the relative abundance at the genus level of the gut microbiota (top 14) of workers of different ages (Table 3). The relative abundance of *Lactobacillus* was only 0.44% 0 dpe and 48.09% 12 dpe. The relative abundances of *Gilliamella* and *Apibacter* were the lowest 0 dpe (both lower than 0.01%), and the relative abundance of *Gilliamella* had increased significantly by 3 dpe and then became stable. The relative abundance of *Apibacter* reached 17.62% 12 dpe and then stabilized. The relative abundances of *Acinetobacter* and *Sphingomonas* were the highest 0 dpe (49.91 and 10.21%, respectively). Interestingly, the relative abundance of both genera decreased significantly after 0 dpe, and although *Acinetobacter* and *Sphingomonas* still occupied a certain niche, their relative abundances in the gut of the workers were not high and tended to be stable. *Snodgrassella* and *Bifidobacterium* are important bacteria in the gut of workers. Their relative abundances were the lowest 0 dpe; the highest relative abundance of *Snodgrassella* was observed 25 dpe (14.28%), and the highest relative abundance of *Bifidobacterium* was observed 7 dpe (11.98%). The relative abundance of bacteria of the genera *Peptostreptococcaceae*, *Escherichia-Shigella*, *Bacteroides*, *Tatumella*, *Cyanobacteria*, *Fusobacterium* and *Gammaproteobacteria* in the gut of workers was higher than 1%, and the relative abundance was significantly different at different ages of *A. cerana* (Figure 2 and Table 3). The abundance of *Peptostreptococcaceae* was lowest 0 dpe and highest 1 dpe (*P* = 0.006). The abundance of *Escherichia-Shigella* was highest 1 dpe and lowest 12 dpe. The abundance of *Bacteroides* was lowest 0 dpe and highest 1 dpe (*P* = 0.016). The abundance of *Tatumella* was lowest 7 dpe and highest 25 dpe. The changes in the abundances of *Cyanobacteria*, *Fusobacterium* and *Gammaproteobacteria* were significantly different; *P-*values were as follows: *P* = 0.019, *P* = 0.005, *P* = 0.012, respectively.

**TABLE 3:** Top 13 abundant genera.

**Genera**	**Different post time (%)**									***P* value**
	**0 dpe^a^**	**1 dpe^b^**	**3 dpe**	**7 dpe^c^**	**12 dpe**	**19 dpe^d^**	**25 dpe**	**30 dpe^e^**	**35 dpe**	
*Lactobacillus*	0.44	14.01	45.67	46.19	48.09	37.67	25.34	12.47	22.88	0.003
*Gilliamella*^bc^	0.01	0.22	20.88	16.91	21.95	30.39	26.86	16.88	40.20	0.011
*Apibacter*^bc^	0.00	0.04	1.64	8.91	17.62	16.39	23.73	20.71	19.63	0.006
*Acinetobacter*^ab^	49.91	0.58	0.21	0.12	0.04	0.07	0.04	0.21	0.04	0.005
*Snodgrassella*	0.00	0.02	0.05	2.28	2.23	2.68	14.28	9.48	4.11	0.005
*Bifidobacterium*	0.04	0.11	3.90	11.98	1.66	3.62	0.75	1.75	1.79	0.026
*Peptostreptococcaceae*^ab^	0.12	11.87	2.70	1.61	0.22	0.57	0.37	4.54	1.86	0.006
*Escherichia-Shigella^bc,cd^*	3.59	6.71	1.45	1.53	0.12	0.31	0.13	1.79	0.51	0.001
*Bacteroides*^ab, bc^	0.12	5.54	1.54	0.73	0.20	0.28	0.20	1.75	0.64	0.016
*Sphingomonas*^ab, bc^	10.24	0.31	0.17	0.06	0.02	0.04	0.02	0.15	0.01	0.002
*Tatumella*	0.24	0.06	0.04	0.01	3.45	1.50	4.25	0.13	0.15	0.007
*Cyanobacteria*	1.23	1.50	1.83	0.77	0.82	0.63	0.09	1.13	0.48	0.019
*Fusobacterium*^ab, bc^	0.01	3.60	0.94	0.56	0.16	0.20	0.17	1.81	0.72	0.005
Others	33.63	51.40	17.67	7.89	3.27	5.42	3.67	17.77	6.64	

*^*ab*^Indicates that the relative abundance 0 dpe and 1 dpe at two developmental stages is significantly different (Student’s *t* test).*

*^*bc*^Indicates that the relative abundance 1 dpe and 7 dpe at two developmental stages is significantly different (Student’s *t* test).*

*^*cd*^Indicates that the relative abundance 7 dpe and 19 dpe at two developmental stages is significantly different (Student’s *t* test).*

Interestingly, we performed statistical analysis on the intestinal flora of bees at different developmental stages, and the results showed that at the phylum and genus levels, there were significant changes in the relative abundance of 19 phyla and 236 genera, respectively. Details of the relative abundances are shown in Supplementary Table 1.

### Beta Diversity of Gut Bacteria

**FIGURE 3:**
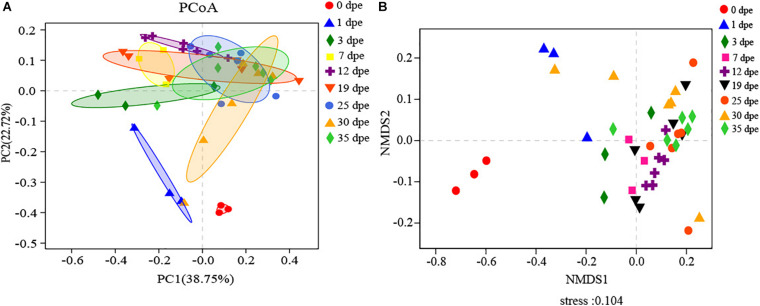
**(A,B)** The horizontal and vertical coordinates represent the two selected principal coordinate components, and the percentage represents the contribution value of the principal coordinate component to the sample composition difference. The scales of the horizontal and vertical axes are relative distances and have no practical significance. Points of different colors or shapes represent samples of different groups. The closer the two sample points are, the more similar the species composition of the two samples will be.

The gut microbiota was analyzed based on the weighted UniFrac distance of a principal coordinates analysis (PCoA) and Bray-Curtis distance of the non-metric multidimensional scaling (NMDS) method for workers at different dpe. PCoA and NMDS analyses revealed that the compositions of the gut microbiota of workers at different dpe were different (Figure 3). In addition, the gut microbiota of workers 0 dpe was significantly separated from that of workers at other dpe, indicating that the composition of the gut microbiota of workers 0 dpe was significantly different from that at other dpe. Next, the gut microbiota of workers 1 dpe and 3 dpe was separated from that at other dpe, indicating that the composition of the gut microbiota of workers 1 dpe and 3 dpe was different from that at other dpe. Although the distances between the 7 dpe, 12 dpe, 19 dpe, 25 dpe, 30 dpe and 35 dpe groups were small, the samples of different dpe groups were clustered in their respective groups. Overall, samples from each group were concentrated in clusters of workers of different ages. In the PCoA, PC1 accounted for 38.75% of the total variance, and PC2 accounted for 22.72% (Figure 3A). In the NMDS, stress = 0.104, which indicated that the grouping and sampling were reliable (Figure 3B).

## Discussion

*A. cerana* and *Apis mellifera*, as the two largest commercial species of bees in China, not only produce an abundance of bee products with huge economic value but also provide pollination services for crops. In addition, these bees exhibit similar life cycles and behaviors, including division of labor. Previous studies have shown that the gut of *Apis mellifera* consists mainly of nine types of bacteria, *Lactobacillus Firm-4*, *Lactobacillus Firm-5*, *Snodgrassella alvi*, *Gilliamella apicola*, *Bifidobacterium*, *Frischella perrara*, *Bartonella apis*, *Parasaccharibacter apium* and Alpha 2.1 (Jeyaprakash et al., 2003; Babendreier et al., 2007; Bottacini et al., 2012; Kwong and Moran, 2013; Philipp et al., 2013). In addition, many published studies have shown that gut microbes in *A. mellifera* undergo dynamic changes at different developmental stages, and there were significant correlations between microbes and host development, aging and social behavior (Dong et al., 2020). However, studies on the gut microbes of *A. cerana* at different stages of host development are rare. This fact is not conducive to the recognition and protection of this important bee species. Therefore, this study used high-throughput sequencing technology to explore the gut microbes of *A. cerana* at different stages of development.

The gut microbiota plays an important role in the health and disease of the host (Raymann and Moran, 2018), and the study of the gut microbiota is of great significance for the protection of bees, important resources, and can further reveal the dynamic succession of the insect gut microbiota. The results showed that the diversity of the gut microbiota changed significantly throughout the life cycle of bees. Interestingly, the diversity of the gut microbiota of *A. cerana* was highest 1 dpe. Previous studies reported that the gut microbiota of honey bees was acquired mainly through social contact (Powell et al., 2014). Stephens et al. (2016) studied the gut microbiota of zebrafish at different developmental stages and found that environmental exposure in early development had the greatest impact on the gut microbiota (Stephens et al., 2016). After pupation of *A. cerana* 0 dpe, the bees were quickly in contact with the hive environment and the older bees, which may be an important reason for the significant increase in gut microbiota diversity 1 dpe. Therefore, environmental exposure has a huge impact on the diversity of the gut microbiota during the development of the host. This phenomenon is true for insects and fish. However, further research is needed to determine whether this theory applies to other animal groups. In addition, the same is true for infants who have a low gut microbiota diversity, which becomes more abundant as the infants grow (Azad et al., 2015). It can be inferred from these results that the transformation of gut microbiota diversity of *A. cerana* is similar to that of humans; it may be a common rule that gut microbiota diversity changes with host development, but further experimental exploration is needed.

In this study, it was found that 0 dpe samples lacked the core microbiota in the gut, which was consistent with previous reports (Yun et al., 2018). The results of this study further support the reliability of previous results. The gut microbiota of *A. cerana* 0 dpe is dominated by Proteobacteria, which are among the major gut microbiota constituents of other insects (Yun et al., 2014). These results indicate that the dominant role of Proteobacteria in the insect gut microbiota may be a distinctive feature of insect gut microbiota composition. Notably, this hypothesis needs to be confirmed by analysis of the gut microbiota of more insect groups. In addition, the composition of the gut microbiota of *A. cerana* and *Anoplophora glabripennis* at the phylum level is similar, with both being dominated by Proteobacteria, Firmicutes, Bacteroidetes and Actinobacteria (Schloss et al., 2006). This relatedness indicates that the gut microbial compositions of *A. cerana* and *A. glabripennis* are highly similar, and we preliminarily speculate that this structure may be the unique composition of the gut microbiota of insects.

The relative abundance of the dominant bacteria of the *A. cerana* gut microbiota at the genus level varied significantly at different developmental stages. At 0 dpe, the main components were *Acinetobacter* and *Sphingomonas*. *Acinetobacter* is an important part of the gut microbiota of many insects and has been found in the gut microbiota of *Pardosa laura*, *Pardosa astrigera*, *Nurscia albofasciata*, *Omphisa fuscidentalis* and other insects (Liu et al., 2016; Hu et al., 2019). *Acinetobacter* assists the host in digesting food and converting nitrogen (Briones-Roblero et al., 2016) and is an important insect gut probiotic. However, after 1 dpe, *Acinetobacter* was quickly replaced by other dominant bacteria, which may be due to the gut microbiota adapting to a more complex environment, as it does 0 dpe, *A. cerana* had not yet come into contact with the environment. *Apibacter* is highly abundant in the gut of *A. cerana* and bumblebees; however, its abundance in the gut of *A. mellifera* is low (Smagghe et al., 2016; Kwong et al., 2018), and *Apibacter* metabolizes mainly monosaccharides and dicarboxylic acids (Kwong et al., 2018). *Apibacter* abundance increased significantly by 7 dpe, indicating that the host began to ingest large amounts of monosaccharides or was on the verge of metabolizing and absorbing monosaccharides. *Lactobacillus*, *Gilliamella*, *Snodgrassella* and *Bifidobacterium* compose the core microbiota of bees and occupy an important niche (Babendreier et al., 2007; Bottacini et al., 2012; Kwong and Moran, 2013). The relative abundances of *Lactobacillus*, *Gilliamella*, *Snodgrassella* and *Bifidobacterium* in the gut were low 0 dpe, and they then rapidly colonized the gut 1-7 dpe. Previous studies have shown that *Lactobacillus* and *Bifidobacteria* can promote the absorption of nutrients and activate the host immune system (Alberoni et al., 2018). Therefore, an increase in the relative abundance of both microbes may indicate rapid development of workers and changes in diet. In addition, *Bifidobacterium* can stimulate the production of hormones by the host, which can affect the development of bees and accelerate the development of workers (Kešnerová et al., 2017). Therefore, the rapid increase in *Bifidobacterium* abundance in the gut of workers may coincide with the peak of *A. cerana* development. *Gilliamella* and *Bifidobacterium* are involved in the degradation of complex polysaccharides (Zheng et al., 2016; Kwong et al., 2018); however, pollen, bee bread and honey contain a variety of complex polysaccharides, and colonization by these two microbes promotes the catabolism of these compounds, indirectly promoting the development of workers. Interestingly, while workers, such as nurse bees, mainly feed larvae and old bees (Seeley, 1982; Crailsheim, 1991, 1992), the feeding material depends mainly on the metabolism of pollen and polysaccharides by these microbes. Therefore, these microbes in the gut of workers may also contribute to changes in the host’s social behavior.

An interesting phenomenon in this study is that the succession of the gut microbiota of *A. cerana* occurs via the constant colonization of core microbiota at different developmental stages and the replacement of non-core microbiota 0 dpe. The colonization of the core microbiota is a dynamic process, but once established the composition of the microbiota is relatively stable.

Notably, although certain negative controls were needed to prevent erroneous results, we lacked these controls, and the absence of these controls may have affected the results of this study to some extent. A negative control was not included for the materials (tubes, swaps, etc.) used in this study; therefore, there is a potential risk of introducing a contaminating sequence. This study mainly focused on the changes in the main dominant microbiota in the gut of workers at different developmental stages. However, a negative control is certainly important for the study of gut microbiota, especially for improved data interpretation (Hornung et al., 2019). Therefore, in future gut microbial-related studies, multiple controls should be adopted to reduce the influence of environmental factors on the conclusions. In addition, to ensure the reliability of the experimental results, it is necessary to add some positive controls such as (1) a positive control for DNA extraction, to ensure that the DNA of the contained organisms can be sufficiently extracted with the method used, and (2) a positive control for sequencing (a pre-extracted DNA mix), to ensure that the sequencing itself did not introduce any errors (Hornung et al., 2019).

In summary, exploration of the colonization characteristics of the gut microbiota in insects is an essential step for further understanding microbiota formation in the animal gut. Here, the composition and abundance of the gut microbiota were determined and quantified comprehensively in workers, and the colonization pattern of the gut microbiota and various genera was further revealed in the comparison across different time points within 35 days after worker pupation. In particular, the colonization characteristics of the gut microbiota of workers were compared with those of other species of animals (mainly vertebrates) based on the overall tendency of microbiota colonization and genera (or species), which revealed several common and characteristic colonization rules between insects and other animals. This study not only deepens our understanding of the colonization pattern of the gut microbiota in workers but also provides useful information for exploring colonization of the gut microbiota in insects and other animals more comprehensively.

## Data Availability Statement

The datasets generated for this study can be found in the raw reads were deposited into the NCBI Sequence Read Archive (SRA) database (Accession Number: SRR9715685-SRR9715699 and SRR9715700-SRR9715726).

## Author Contributions

Z-XD wrote the article on data analysis. H-YL, Y-FC, and Q-HT took samples. JG designed the experiment. All authors contributed to the article and approved the submitted version.

## Conflict of Interest

The authors declare that the research was conducted in the absence of any commercial or financial relationships that could be construed as a potential conflict of interest.

## Footnotes

1 http://drive5.com/uparse/

2 http://rdp.cme.msu.edu/
