# Supplementary figures and images for: The Succession of the Gut Microbiota in Insects: A Dynamic Alteration of the Gut Microbiota During the Whole Life Cycle of Honey Bees (Apis cerana)
Source: Front Microbiol. 2021 Apr 14;12:513962. doi: 10.3389/fmicb.2021.513962 (PMC8079811; doi:10.3389/fmicb.2021.513962)

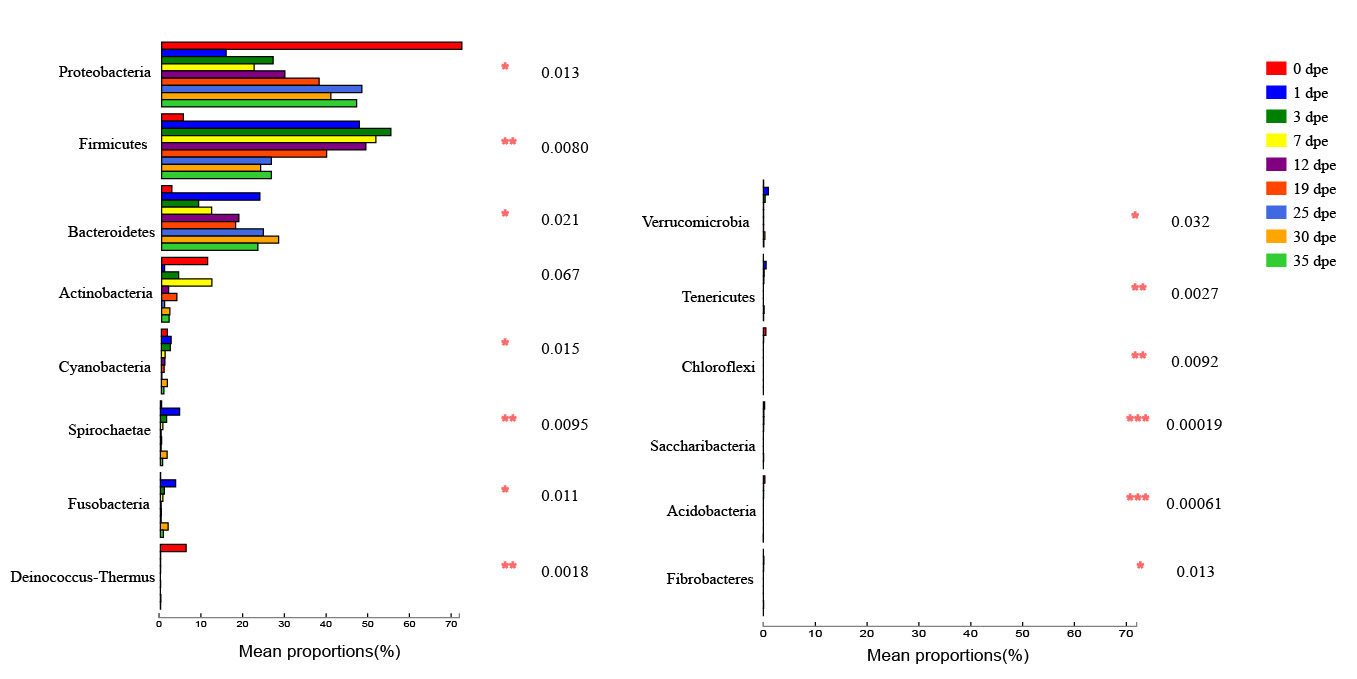

Supplement: Supplementary file 2 [file Image_1.JPEG]
